# Content-Adaptive Reversible Data Hiding with Multi-Stage Prediction Schemes

**DOI:** 10.3390/s25196228

**Published:** 2025-10-08

**Authors:** Hsiang-Cheh Huang, Feng-Cheng Chang, Hong-Yi Li

**Affiliations:** 1 Department of Electrical Engineering, National University of Kaohsiung, Kaohsiung City 811726, Taiwan; hchuang@nuk.edu.tw (H.-C.H.);; 2 Department of Computer Science and Information Engineering, Tamkang University, New Taipei City 251301, Taiwan

**Keywords:** reversible data hiding, content inherent characteristics, weighted average prediction, difference histogram, quadtree decomposition

## Abstract

With the proliferation of image-capturing and display-enabled IoT devices, ensuring the authenticity and integrity of visual data has become increasingly critical, especially in light of emerging cybersecurity threats and powerful generative AI tools. One of the major challenges in such sensor-based systems is the ability to protect privacy while maintaining data usability. Reversible data hiding has attracted growing attention due to its reversibility and ease of implementation, making it a viable solution for secure image communication in IoT environments. In this paper, we propose reversible data hiding techniques tailored to the content characteristics of images. Our approach leverages subsampling and quadtree partitioning, combined with multi-stage prediction schemes, to generate a predicted image aligned with the original. Secret information is embedded by analyzing the difference histogram between the original and predicted images, and enhanced through multi-round rotation techniques and a multi-level embedding strategy to boost capacity. By employing both subsampling and quadtree decomposition, the embedding strategy dynamically adapts to the inherent characteristics of the input image. Furthermore, we investigate the trade-off between embedding capacity and marked image quality. Experimental results demonstrate improved embedding performance, high visual fidelity, and low implementation complexity, highlighting the method’s suitability for resource-constrained IoT applications.

## 1. Introduction

With the proliferation of image-capturing and display-enabled Internet of Things (IoT) devices, ensuring the authenticity and integrity of visual data has become increasingly critical. Information security has become an important issue across applications from medical imaging to intellectual property protection [1]. One of the effective means of security schemes is data hiding. Traditional techniques in data hiding, while effective in concealing information, typically result in irreversible modifications to host media, making them unsuitable for applications that require complete data recovery. This limitation has driven significant research interests in reversible data hiding (RDH) techniques, which enable both the perfect extraction of hidden information and perfect restoration of original media [2,3,4].

Reversible data hiding addresses the fundamental challenge of preserving data integrity while enabling secure information transmission. It is an important topic in this research area, with the following current trends. Authors applied two images with Hamming codes and arithmetic coding with the exploiting modification direction (EMD) and difference alteration techniques for reversible data hiding [5,6]. Authors chose to enhance histogram-based RDH techniques with least significant bit (LSB) replacement and block-wise histogram-shifting methods [7,8]. Authors applied RDH with the concept of secret sharing by proposing an enhanced algorithm for the applications to shard JPEG images [9,10]. In [11], authors extended RDH applications to intelligent transport applications. These trends may enrich the applications, along with the enhancement in algorithm design.

Unlike conventional steganographic methods, RDH techniques must satisfy stringent lossless recovery requirements, making them particularly valuable in sensitive applications such as legal documentation, where permanent alterations could compromise critical processes. However, the reversibility constraint introduces significant technical challenges in achieving optimal trade-offs among the metrics for assessing the performance of RDH algorithms [12,13,14]. Important metrics for RDH include the quality of the marked image, implying the differences between original and marked images, and the capacity for embedding, implying the number of bits hidden into the original image. Because the quality of the marked image highly depends on the original image, content characteristics of the original one may help to enhance the performance, and this is the main contribution for this paper.

Current RDH approaches encompass three main paradigms. Firstly, histogram shifting methods offer good visual quality but limited capacity [15,16,17]. Secondly, difference expansion techniques provide a higher embedding capacity with potential artifacts or degraded quality [18,19,20]. Finally, prediction error expansion strategies represent current state-of-the-art performance [21,22,23,24], with the concepts from the first and second paradigms. Despite advances, existing methods face critical limitations, including capacity ceilings from single-stage embedding, fixed prediction models struggling with diverse image characteristics, and uniform processing strategies that fail to exploit spatial heterogeneity in natural images [25,26,27].

In this paper, we present a unified framework integrating weighted average prediction with different embedding strategies with the consideration of image characteristics: namely, subsampling and quadtree decomposition. The primary contributions include the development of a weighted average predictor achieving substantial improvements over conventional methods, the implementation of spatial processing approaches, the introduction of adaptive embedding mechanisms, and comprehensive evaluation demonstrating significant performance gains for grayscale and color images [28,29,30].

## 2. Prediction-Based Reversible Data Hiding Schemes

### 2.1. Fundamentals of Reversible Data Hiding

In this paper, we apply the image prediction method to produce the predicted images, calculate the differences between the original and predicted ones, and utilize the difference values for reversible data hiding. Before the description of our algorithm, we first address the fundamental concepts and notations for reversible data hiding.

**Figure 1:**
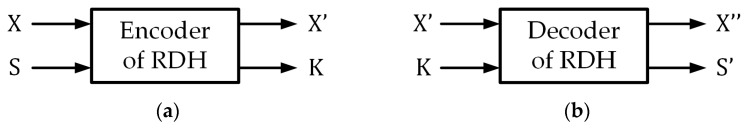
Block diagrams of reversible data hiding (RDH). (**a**) Encoder. (**b**) Decoder.

In Figure 1, we provide the block diagrams for reversible data hiding. Figure 1a denotes the encoder of RDH. Suppose we have the original image X and the secret S. Original images can be grayscale or color images with the size of M×N, while S is presented with binary format with the length of C bits. The secret S should be binary bitstream, and it may contain the contents determined by authors. Here, we apply random bitstreams of length C bits, with 50% of bit 0’s and 50% of bit 1’s, for reversible data hiding. With a reversible data hiding algorithm, devised by researchers, the secret S is embedded into the original X, and the marked image $X^{\prime }$ is produced. Meanwhile, the secret key K for decoding is also determined. In contrast, Figure 1b denotes the decoder of RDH. We can easily notice that the outputs of Figure 1a are identical to their counterparts of the inputs of Figure 1b. With the provision of the secret key K, we have the outputs $X^{\prime \prime }$ and $S^{\prime }$ at the decoder output, which can be regarded as the separation of $X^{\prime \prime }$ and $S^{\prime }$ from $X^{\prime }$. In practice, the secret key K, which consists of the rotation angles and predictor weights, can be transmitted separately from the marked image and protected using standard cryptographic mechanisms (e.g., symmetric or asymmetric encryption, secure channels). Thus, the protection of K is orthogonal to the proposed RDH framework.

There are metrics for assessing the performance of RDH algorithms. The most important metric is reversibility. It should be examined in two aspects. Firstly, the original image X in Figure 1a should be identical to decoded image $X^{\prime \prime }$ in Figure 1b. Secondly, the embedded secret S in Figure 1a should be identical to the extracted secret $S^{\prime }$ in Figure 1b. We can calculate the mean squared error (MSE) between X and $X^{\prime \prime }$. If the MSE equals 0, it implies that they are identical. Note that the MSE between the input image X and the output one $X^{\prime }$ can be represented with

(1) $$MSE=\frac{1}{M\cdot N}\sum_{i=1}^{M}\sum_{j=1}^{N}{\left(X^{\prime }\left(i,j\right)-X\left(i,j\right)\right)}^{2}.$$

It is easily observed that MSE≥0. In addition, we can use the exclusive-NOR (XNOR) gate to check whether there are differences between S and $S^{\prime }$. With the examination of the above procedures, we can guarantee the reversibility of an RDH algorithm.

In addition, marked image quality and embedding capacity play important roles for the assessment of performances. In Figure 1a, the marked image $X^{\prime }$ is expected to be presented as similar to the original image X. The peak signal-to-noise ratio (PSNR) and the structural similarity (SSIM) are commonly employed measures, and larger PSNR or SSIM values are expected. PSNR is an objective measure with the unit of decibel (dB), which directly corresponds to MSE. Next, PSNR can be calculated with

(2) $$PSNR=10\cdot \log_{10}\left(\frac{255^{2}}{MSE}\right)(\mathrm{dB}).$$

SSIM relates to the subjective rating of image quality, which may work together with PSNR for performance assessments. Moreover, the bitstream length C would be expected to be as long as possible. Due to the fact that data embedding introduces errors to form a marked image, embedding more amounts of bits leads to larger errors in the marked image, which implies that there is more serious degradation in the marked image. To normalize the comparisons, embedding capacity could be represented by the ratio between the amount of embedding bits and the size of the original image, or $\frac{C}{M\times N}$, with the unit of bit per pixel (bpp). Therefore, enhanced embedding capacity, acceptable quality after data hiding, and reasonable amount of side information would be required for the design of an algorithm.

### 2.2. Prediction-Based Reversible Data Hiding

Major limitations for conventional reversible data-hiding techniques would be the limited amount of embedding capacity [2] or the degraded quality of the marked image [3,4]. For the histogram-based techniques, guaranteed quality of the marked image with the condition of a limited amount of capacity can be observed. With [2], the maximum of MSE between marked and original images in Equation (1) is 1, and then with the calculation in Equation (2), PSNR should be at least 48.13 dB, implying a guaranteed quality of the marked image. In contrast, for difference-expansion-based techniques, an enhanced amount of capacity with much degraded quality can be monitored.

One effective way to increase the embedding capacity while keeping the marked image quality is the use of prediction schemes [24,25]. The concept of applying prediction schemes comes from the integration of the advantages of histogram-based and difference-expansion-based techniques. At the encoder, a predicted image can be produced from the original image X with the provision of weight factors, which may serve as K in Figure 1a, and the difference values between the two can be recorded. Then, the difference histogram can be prepared, and secret data S can be embedded with the intentional movement of some portions of the difference histogram, which leads to the modified difference values. Next, by adding back the modified difference values to the original image, the marked image $X^{\prime }$ can be acquired. At the decoder, with the provision of weight factors, which may serve as K in Figure 1b, the luminance values in the predicted image can be calculated from the marked image $X^{\prime }$. Secret information $S^{\prime }$ can be extracted by moving back some portions of the difference histogram, and then the difference values can be added back to the marked image $X^{\prime }$ to produce the output image $X^{\prime \prime }$ in Figure 1b. We expect that both images X and $X^{\prime \prime }$ are identical, and both secrets S and $S^{\prime }$ are identical, to keep the reversibility.

**Figure 2:**
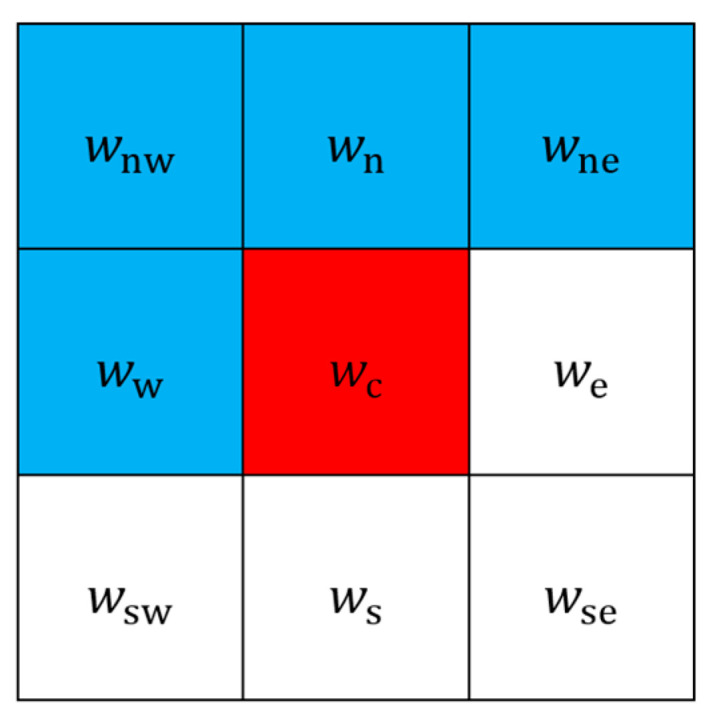
Weight factors for pixel value prediction. Subscripts imply the directional relationships to the central factor $w_{c}$. The four factors in blue are employed for prediction.

We employ calculations with weighted average prediction to produce a predicted image from the original one. In Figure 2, locations in blue are served as a mask for performing prediction. The four weight factors in the upper-left (northwest), upper (north), upper-right (northeast), and left (west) side, represented by $w_{nw}$, $w_{n}$, $w_{ne}$, and $w_{w}$, are trained beforehand. Due to the fact that predicted and original images should look similar, we set the four weights to be positive integers. Predicted luminance value at the center location, or the one in red, can be calculated with a weighted average from the four weight factors in the mask with Equation (3).

(3) $$X_{p}\left(i,j\right)=round\left(\frac{w_{nw}X\left(i-1,j-1\right)+w_{n}X\left(i-1,j\right)+w_{ne}X\left(i-1,j+1\right)+w_{w}X\left(i,j-1\right)}{w_{nw}+w_{n}+w_{ne}+w_{w}}\right).$$

By applying the computation when all four weighted pixels are available, the predicted pixels can be determined. With this manner, if we take the original image with the resolution of 1024×1024 pixels, there will be 1022×1023 predicted pixels produced with Equation (3). The first and the last column, and the first row in the predicted image, are identical to their counterparts in the original image, keeping the same image size between the original and predicted images. With Equation (3), we can present all the pixels in the predicted image as $X_{p}$. 

Next, we can calculate the difference between X and $X_{p}$, and then generate the difference histogram.

(4) $$D\left(i,j\right)=X\left(i,j\right)-X_{p}\left(i,j\right).$$

**Figure 3:**
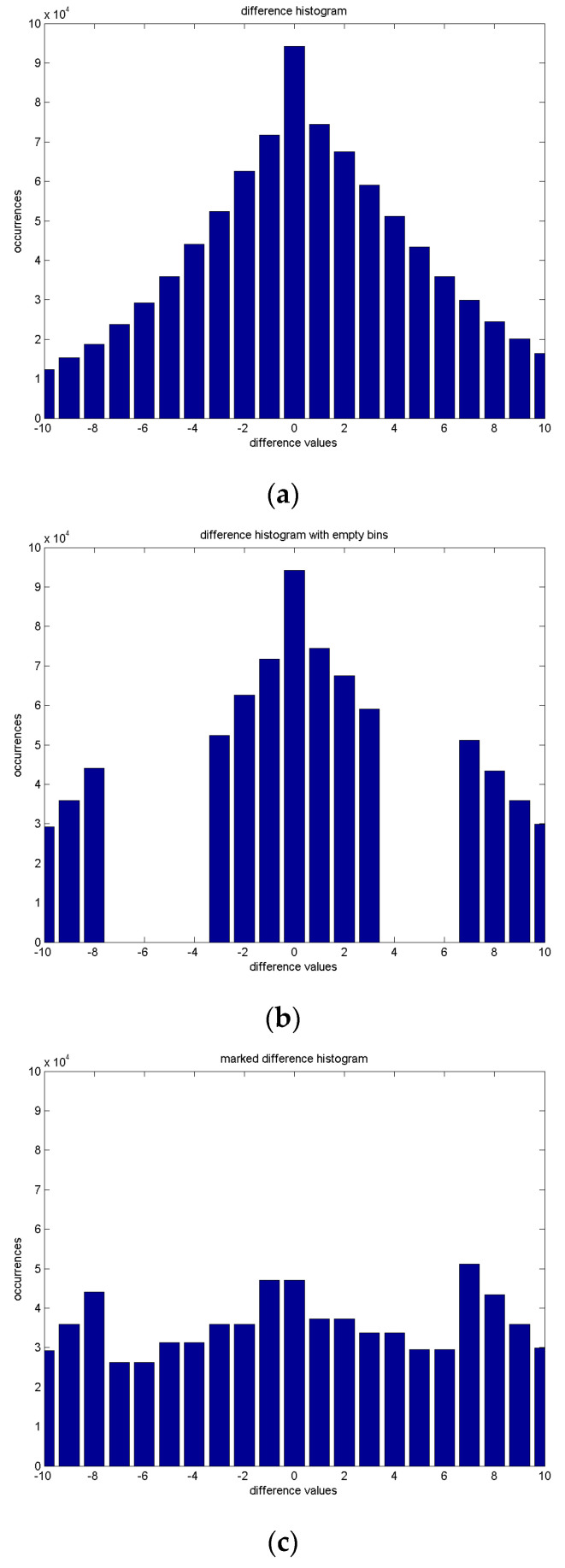
Illustration of data embedding with prediction-based scheme. Horizontal and vertical axes denote the difference values and occurrences, respectively. (**a**) Difference histogram between original X and predicted image $X_{p}$. (**b**) Intentional emptying of certain bins. (**c**) Difference histogram after data embedding.

Here, $D\left(i,j\right)$ can be regarded as the difference image, with the difference values ranging from −255 to 255. The smallest and largest difference values in our example are −243 and 232, respectively. We observe that predicted and original images look similar; hence, a large portion of the difference value concentrates are around 0. Due to the concentration of difference values around zero, we display the portion between −10 and 10 for demonstration purposes in Figure 3a.

For data embedding, we choose the embedding level (EL), which is a positive integer, and keep the differences between EL and −EL intact. Except for the portions between EL and −EL in the difference histogram, we call the positive ones the right tail, and the negative ones the left tail. We intentionally move the right tail rightward and move the left tail leftward to produce the empty bins in Figure 3b. With the selected EL, we can obtain an emptied difference histogram with Equation (5):

(5) $$D^{\prime }\left(i,j\right)=\left\{\begin{array}{cc} D\left(i,j\right)+EL, & if\;D\left(i,j\right)>EL;\\ D\left(i,j\right), & \;\;if\;\left|D\left(i,j\right)\right|\leq EL;\\ D\left(i,j\right)-EL-1, & \;\;\;if\;D\left(i,j\right)<-EL. \end{array}\right.$$

Then, the secret bitstream S can be embedded into the empty bins to produce Figure 3c. With the embedding of bitstreams, or the composition of random bits, we can obtain a marked difference histogram for the demonstration in Equation (6).

(6) $$D^{\prime \prime }\left(i,j\right)=\left\{\begin{array}{cc} D^{\prime }\left(i,j\right), & \;\;if\;D^{\prime }\left(i,j\right)>2\cdot EL;\\ 2\cdot D^{\prime }\left(i,j\right)-b, & if\;\left|D^{\prime }\left(i,j\right)\right|\leq EL;\\ D^{\prime }\left(i,j\right), & \;\;\;\;\;\;\;\;\;\;\;\;\;if\;D^{\prime }\left(i,j\right)<-2\cdot EL-1. \end{array}\right.$$

Here, b denotes the secret bit to be embedded. The value of b=0 or b=1 is applied to Equation (6) for data embedding.

We take Figure 3 as an instance. In Figure 3, the horizontal axis denotes the difference values in the histogram, and the vertical axis denotes the occurrences. Figure 3a displays the difference histogram between the original and predicted images, clipped to the range between −10 and 10 for better representation. Figure 3b denotes the emptied bins with Equation (5), for embedding with the condition that EL=3. It implies that there are seven bins, ranging from −3 to 3, or from −EL to EL, that are ready to be modified for data embedding. For embedding binary data, a total of 7×2=14 bins should be prepared. Aside from the existing seven bins that are constrained by EL=3, we need to prepare for an additional seven empty bins, as pointed out in Equation (5) to generate $D^{\prime }\left(i,j\right)$. Without the loss of generality, we set four empty bins in the negative part, or −4 to −7, and three empty bins in the positive part, or 4 to 6, as depicted in Figure 3b. The positive difference values larger than EL, called the right tail, are intentionally moving rightward by three. At the same time, the negative difference values smaller than −EL, called the left tail, are intentionally moving leftward by four. These steps produce the emptied difference histogram in Figure 3b. There might be a very low probability that the difference values may reach both extremes, 255 or −255. The location map [4] should be provided for decoding, to keep the reversibility. In Figure 3a, the extremes in the difference histogram are −243 on the lefthand side and 232 on the righthand side. Therefore, no overflow problem can be observed.

After that, for embedding the bit b one at a time, we follow Equation (6) for data embedding by re-assigning the difference values to produce the marked difference histogram $D^{\prime \prime }\left(i,j\right)$ in Figure 3c.

Finally, the modified difference values are added back to the predicted image to produce the marked image $X^{\prime }$

(7) $$X^{\prime }\left(i,j\right)=D^{\prime \prime }\left(i,j\right)+X_{p}\left(i,j\right).$$

Figure 3 displays an illustration for prediction-based reversible data hiding. With prediction-based techniques for reversible data hiding, we can expect to embed a much higher capacity for the acceptable degradation of the marked image.

### 2.3. Comparisons of Different Approaches in Reversible Data Hiding

For the better understanding of the readers, we are going to provide a table to make comparisons between different approaches for reversible data hiding. Table 1 provides comparisons for RDH, including a histogram-based scheme and a difference expansion scheme, as we stated in Section 2.1, and a conventional prediction and difference histogram scheme, as we noted in Section 2.2. The proposed scheme, or the content-based prediction and difference histogram scheme, inherited the characteristics from the three types, with the consideration of applying image contents to look for better performances in reversible data hiding. We expect the readers to have an overview of the major schemes in RDH in Table 1.

## 3. Proposed Schemes

### 3.1. Enhancements of Embedding Capacity with Content Characteristics

We are going to explore the inherent characteristics of image content for reversible data hiding. In order to look for the increase in embedding capacity, we follow the schemes in [25]. With the method described in Section 2, it can be regarded as the embedding procedure for the first time. To meet with the following notations, we rename $X^{\prime }$ in Figure 1a as $X_{1}^{\prime }$ containing the secret $S_{1}$. We then rotate $X_{1}^{\prime }$ clockwise by 90°, perform image prediction, and modify the difference histogram to hide another set of secret $S_{2}$ and obtain the marked image $X_{2}^{\prime }$. Next, we can rotate $X_{2}^{\prime }$ by 90°, hide secret $S_{3}$ with the procedures above, and obtain the marked image $X_{3}^{\prime }$. This procedure can be executed a number of times, as determined by users, and we call it multi-stage reversible data hiding. For decoding, we perform the operations in the reverse order for the extraction of the secrets stage by stage, and finally restore the original image at the decoder.

To extend the application for prediction-based reversible data hiding of the whole image, we may consider that the original image is composed of blocks with fixed or variable sizes. Each block may present different characteristics of the original image, and different parameters may be applied for data hiding in order to look for enhanced performances. Here, we employ the subsampling concept to acquire four fixed-sized blocks, and the size of each block corresponds to the quarter-sized original image. Moreover, we apply quadtree decomposition to obtain square blocks of different sizes. These blocks may correspond to different content characteristics, and then we can apply prediction-based reversible data hiding accordingly. Different sets of weight factors, which serve as side information for decoding, can be trained to embed enhanced amounts of capacities, corresponding to blocks in different positions to the original image.

In order to provide more flexibility for data hiding based on the inherent characteristics of the image, with the embedding procedure in Section 2, blocks can be rotated clockwise by one of the four settings, i.e., 0°, 90°, 180°, or −90° (or 270°), in order to generate different weight factors. The rotated angle can be represented by two-bit strings, and it also serves as the side information for decoding. The four rotation angles for block rotation are easy for implementation and provide more flexibility to explore the inherent characteristics. With our implementation, we assign the weights $\left(w_{nw},\;w_{n},\;w_{ne},\;w_{w}\right)$ to be integers between 1 and 15 in Equation (3), which can also be represented by four-bit strings for each weight. The best solution for weight factors can be trained with an exhaustive search by choosing the maximum of the peak value in the difference histogram, among all the combinations between the selected blocks in the predicted and original images. The maximum of the difference histogram can be regarded as the cost function for training. With an exhaustive search, there will be $15^{4}=50625$ combinations. When we use Equation (3) for the first time, it implies that the original image is rotated by 0°, and the first set of weights $\left(w_{nw1},\;w_{n1},\;w_{ne1},\;w_{w1}\right)$ are applied to produce the first predicted block. The first set of secret information is embedded subsequently into all blocks to form the first marked image. Next, the first marked image can be rotated clockwise by one of the four rotation angles determined by users, and the second set of weights $\left(w_{nw2},\;w_{n2},\;w_{ne2},\;w_{w2}\right)$ are applied to produce the first predicted image. The data-embedding procedures can be executed to obtain the second marked image. By following the same manner, data embedding can be performed a number of times, as determined by the user.

### 3.2. Data Hiding Scheme with Image Subsampling

**Figure 4:**
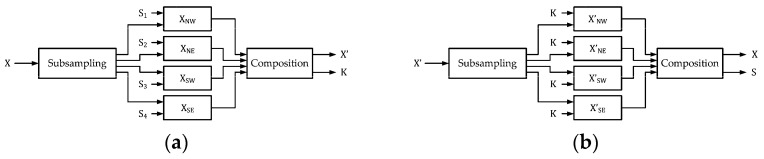
Block diagrams of reversible data hiding with subsampling. (**a**) Encoder. (**b**) Decoder.

Inspired by the prediction-based reversible data hiding scheme, we may divide the original image into four equal-sized blocks directly for data hiding. Each block can be treated as a small image for the execution of data hiding procedures. Corresponding to Figure 1a and Figure 1b, we present reversible data hiding with subsampling with corresponding notations in Figure 4a and Figure 4b, respectively. For the comparisons of notations in Figure 1a and Figure 4a, we set the representation of the secret to be $S=S_{1}\cup S_{2}\cup S_{3}\cup S_{4}$. Notations in Figure 1b and Figure 4b can be directly compared.

The multi-stage rotation method [25] demonstrates enhanced embedding performance and ease of implementation over a conventional prediction-based scheme. It comes from progressive rotations of multiples of 90° of the image, combined with the repeated use of a weighted average predictor in Equation (3). We may randomly choose one of the four rotation angles and train the weights for reversible data hiding with this approach. This subsampling procedure requires the input image area to be square, as the four sub-images are formed by reorganizing pixel positions into equally sized blocks.

**Figure 5:**
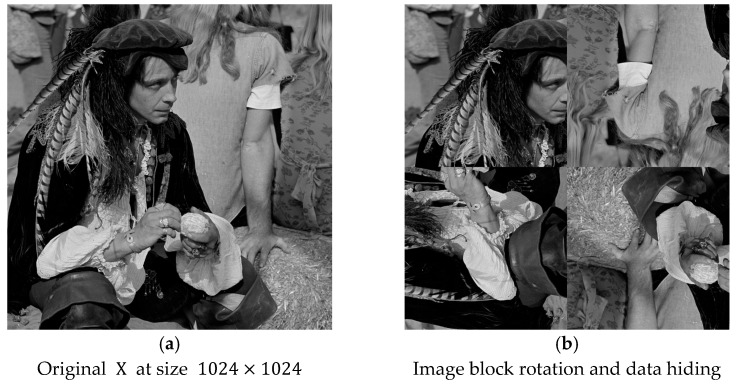
Illustration of data embedding with the image splitting scheme. (**a**) Original image X in size 1024×1024. (**b**) Intentional rotation of four image blocks.

Here is one illustration to demonstrate the concept of the image-splitting scheme performed. The original image male has a size of 1024×1024 in Figure 5a, and we can easily divide it into four equal-sized blocks with sizes of 512×512. The four divided blocks are placed in the upper-left, upper-right, lower-left, and lower-right positions. We can randomly assign the rotation angles, rotated clockwise, with multiples of 90°. As we can see from Figure 5b, the four rotation angles are recorded with an array (0°, 180°, −90°, 180°). After rotation, we train the weight factors and perform prediction-based reversible data hiding to the four blocks independently. This is called the first stage. After the completion of data hiding at the first stage, we can repeatedly perform the same procedures a number of times, as determined by users. Finally, in order to have the normal display of the marked image, after completion of data embedding, each block should be rotated back to 0° to form the marked image.

**Figure 6:**
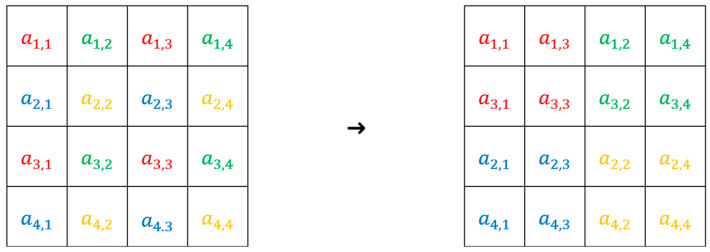
Demonstration of the concept of subsampling for 4×4 image. Original image is composed of luminance values with different colors at certain positions. With subsampling, luminance values with the same color are grouped together.

Furthermore, with the extension to the direct splitting of the original image, we may follow the subsampling concept to perform data hiding. Subsampling refers to the process of reducing the number of pixels used to represent an image, effectively creating a lower-resolution version. In Figure 6, suppose that the original image has a size of 4×4 on the lefthand side, and we use four colors to present specific positions. After subsampling, pixels of the same color are placed in the designated positions on the righthand side. Namely, the red, green, blue, and orange subsampled images are placed at the upper left, upper right, lower left, and lower right positions, respectively. We can apply prediction-based reversible data hiding in Section 2 to each subsampled image independently.

**Figure 7:**
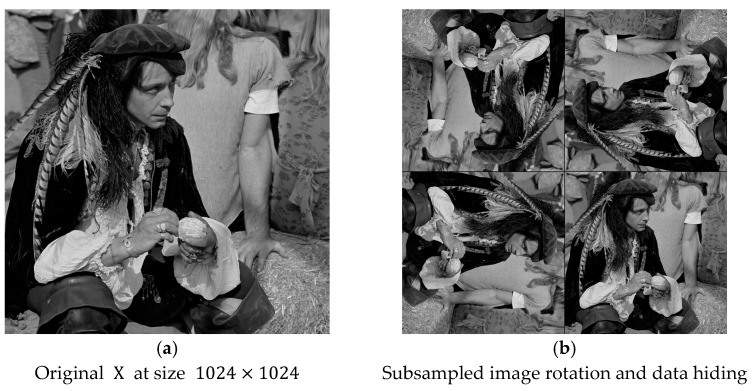
Illustration of data embedding with subsampling-based prediction scheme. (**a**) Original image X at size 1024×1024. (**b**) Intentional rotation of subsampled images.

Figure 7 shows the example for subsampling. Figure 7a is the original image, which is the same as the one in Figure 5a. With the subsampling in Figure 6, we have four subsampled images that look similar in Figure 7b. By following in a similar manner with data hiding corresponding to Figure 3b, in Figure 7b, the four rotation angles are recorded with the array (180°, −90°, 90°, 0°). We can follow procedures in Section 2 for data hiding. For the better comprehension of this scheme, we provide an example in detail in Section 4.1.

### 3.3. Data Hiding Scheme with Quadtree Decomposition

**Figure 8:**
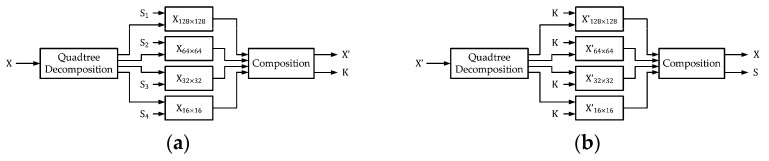
Block diagrams of reversible data hiding with quadtree decomposition. (**a**) Encoder. (**b**) Decoder.

Quadtree decomposition segments the original image into smaller square blocks with different sizes, based on the smoothness or activeness of inherent characteristics [27]. We can apply the data embedding procedure in Section 2 to each block. Due to the nature of quadtree partitioning, the side length of the input image area should be a power of two, so that the recursive block splitting can be applied without a remainder. Corresponding to Figure 1a and Figure 1b, we present reversible data hiding with quadtree decomposition, with corresponding notations in Figure 8a and Figure 8b, respectively. For the comparisons of notations in Figure 1a and Figure 8a, again, we set the representation of the secret to be $S=S_{1}\cup S_{2}\cup S_{3}\cup S_{4}$. Notations in Figure 1b and Figure 8b can be directly compared.

**Figure 9:**
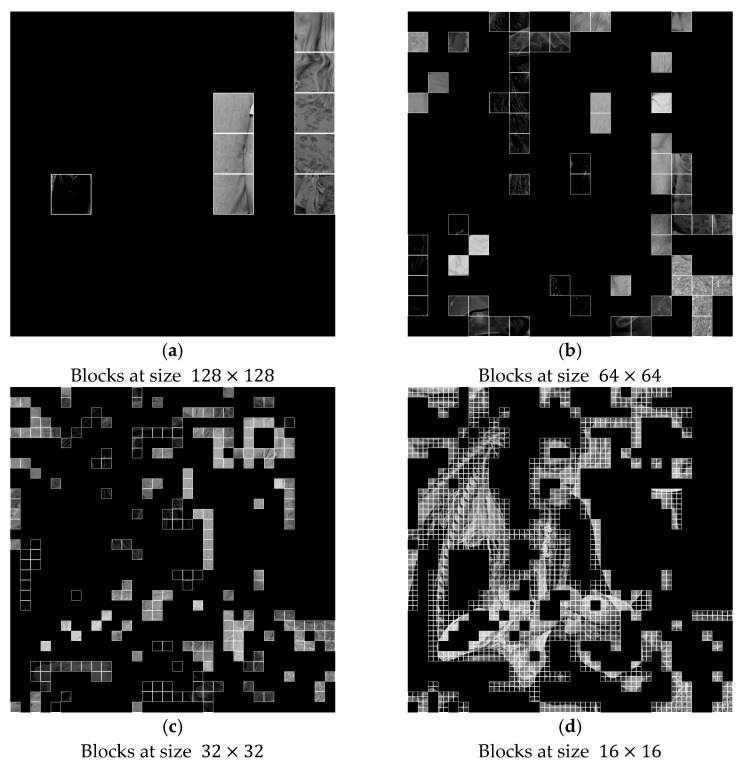
Illustration of data embedding, with quadtree-based prediction scheme. (a) Blocks at size 128×128. (**b**) Blocks at size 64×64. (**c**) Blocks at size 32×32. (**d**) Blocks at size 16×16.

Figure 9 displays an example for quadtree decomposition. Again, we take the original image male with a size of 1024 × 1024, as shown in Figure 5a or in Figure 7a. Then, we can partition the original image into blocks of four sizes: 128×128 in Figure 9a, 64×64 in Figure 9b, 32×32 in Figure 9c, and 16×16 in Figure 9d, respectively. Considering the ease of implementation for reversible data hiding, blocks of the same size correspond to one specific set of weight factors and the rotation angle to form the first stage of data embedding. It can be performed a number of times, as determined by the user. These parameters may follow the inherent characteristics of the original image for enhanced performances. For a better comprehension of this scheme, we provide an example in detail in Section 4.2.

### 3.4. Complexity Analysis

The computational complexity of the proposed scheme is analyzed with respect to the image size N×N. The main steps are summarized below:

1. **Prediction and error computation**: For each pixel, the prediction from four neighboring pixels is calculated and the prediction error is obtained. This requires visiting all pixels once, leading to $O\left(N^{2}\right)$.
2. **Histogram construction and shifting**: The prediction-error histogram is constructed in a single pass over all pixels, and the histogram shifting and embedding are also performed in linear time, i.e., $O\left(N^{2}\right)$.
3. **Multi-stage embedding**: If *s* embedding stages are performed (each involving a 90° rotation and re-embedding), the complexity scales linearly with the number of stages, yielding $O\left(s\cdot N^{2}\right)$.
4. **Weighted predictor training**: Because the embedded payload at stage t changes the error distribution for stage $\left(t+1\right)$, the four integer weights must be re-optimized for every stage and for every (image, payload) pair. Using an exhaustive search for integers over $\left[1,\;15\right]$ for each weight yields $15^{4}$ candidates. Evaluating each candidate entails a linear scan to obtain the histogram peak/criterion, so the training cost per stage is $O\left(15^{4}\cdot N^{2}\right)$. Over s stages, the total training cost becomes $O\left(s\cdot 15^{4}\cdot N^{2}\right)$.
5. **Quadtree partitioning**: The quadtree segmentation involves computing local variances and recursively splitting blocks. Each pixel is visited only a constant number of times across the levels, so the overall complexity is $O\left(N^{2}\right)$.
6. **Side information handling**: The generation and embedding of side information (rotation angles, weights, block sizes) require a negligible amount of time compared to the above steps.

Based on the above analysis, we observe that the encoding complexity is dominated by the weighting-factor training, i.e., $O\left(s\cdot 15^{4}\cdot N^{2}\right)$. In the decoding phase, no such training is required, so the complexity is $O\left(s\cdot N^{2}\right)$. In practical application scenarios, the number of stages, *s*, is typically very small and can be regarded as a bounded constant. Consequently, the per-image decoding cost is $O\left(N^{2}\right)$, i.e., linear in the number of pixels. For the encoding side, although the exhaustive search makes the theoretical bound higher, the exponential factor is fixed and does not grow with N. Thus, in asymptotic terms, the algorithm still scales quadratically with the image size.

## 4. Two Examples

In this section, we present two examples to employ content-adaptive characteristics of original images with image subsampling and quadtree decomposition. These examples can directly extend to the simulations in Section 5 with statistical analyses and comparisons.

### 4.1. Example with Image Subsampling

Here is an example to show the data-embedding procedures with the subsampling concept and the block diagram in Figure 4. We take the color image F16, with the size of 512×512, as an instance. With the ease of implementation and without loss of generalization, we transform the 512×512 color image into a grayscale image $\left(X\right)$, with the linear combination of the red $\left(R\right)$, green $\left(G\right)$, and blue $\left(B\right)$ components [29,30].

(8) X=0.2989×R+0.5870×G+0.1140×B.

**Figure 10:**
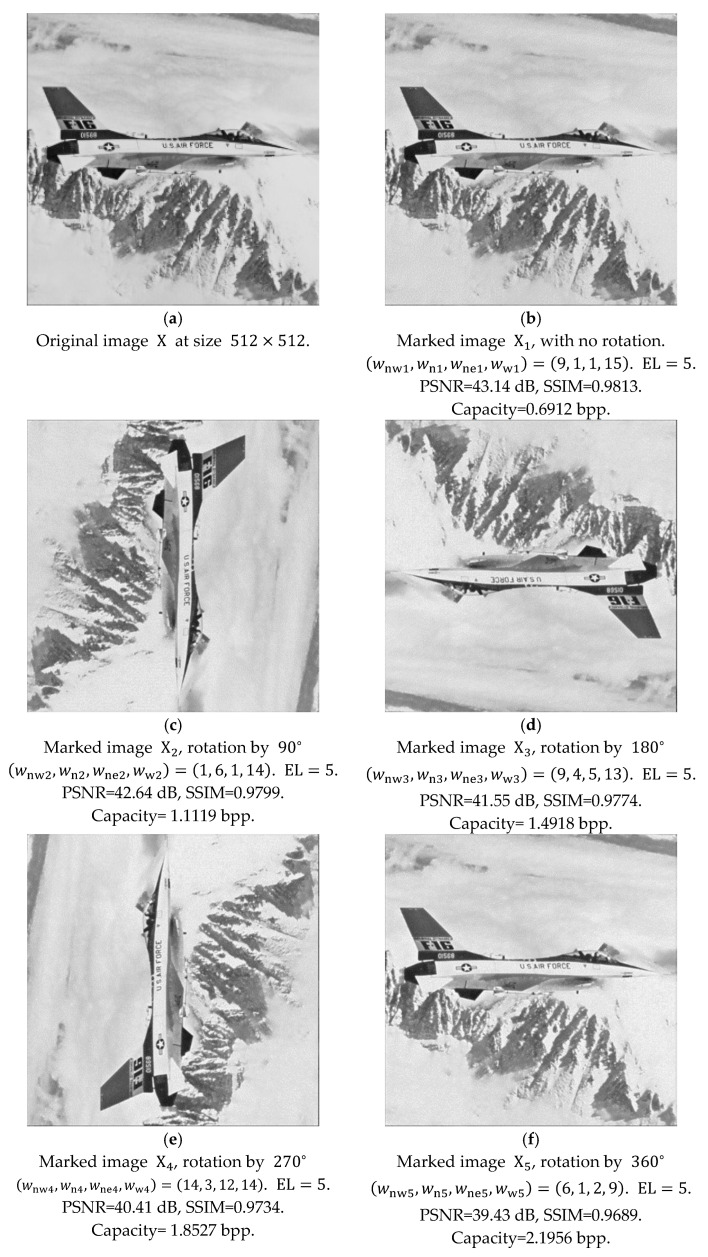
Illustration of data embedding, with multi-stage rotation. (**a**) Original image X at size 512×512. (**b**) Rotation by 0°. (**c**) Rotation by 90°. (**d**) Rotation by 180°. (**e**) Rotation by 270°. (**f**) Rotation by 360°.

In Figure 10a, we have the gray-level image F16 with the calculation of Equation (8). For the first embedding stage, we can calculate the weight factors with the condition of the largest peak of the difference histogram among all 50625 combinations. After obtaining the weight factors of $\left(9,\;1,\;1,\;15\right)$ for the whole image, we obtain the difference values and produce the difference histogram by following the notations in Equation (5). We choose EL=5 and embed one bit at a time, meaning that the difference values are changed with Equation (9).

(9) $$D^{\prime }\left(i,j\right)=\left\{\begin{array}{cc} D\left(i,j\right)+5, & if\;D\left(i,j\right)>5;\\ D\left(i,j\right), & \;\;if\;\left|D\left(i,j\right)\right|\leq 5;\\ D\left(i,j\right)-6, & \;\;if\;D\left(i,j\right)<-5. \end{array}\right.$$

Note that for a better understanding of Equation (9), we use the value of EL directly. When we replace Equation (9) with the notation of EL and embed one bit, it becomes Equation (10), which is identical to Equation (5).

(10) $$D^{\prime }\left(i,j\right)=\left\{\begin{array}{cc} D\left(i,j\right)+EL, & if\;D\left(i,j\right)>EL;\\ D\left(i,j\right), & \;\;if\;\left|D\left(i,j\right)\right|\leq EL;\\ D\left(i,j\right)-EL-1, & \;\;if\;D\left(i,j\right)<-EL. \end{array}\right.$$

For embedding one secret bit, b, the marked difference values should be changed into Equation (11), which is identical to Equation (6).

(11) $$D^{\prime \prime }\left(i,j\right)=\left\{\begin{array}{cc} D^{\prime }\left(i,j\right), & \;\;\;if\;D^{\prime }\left(i,j\right)>2\cdot EL;\\ 2\cdot D^{\prime }\left(i,j\right)-b, & if\;\left|D^{\prime }\left(i,j\right)\right|\leq EL;\\ D^{\prime }\left(i,j\right), & \;\;\;\;\;\;\;\;\;\;\;\;if\;D^{\prime }\left(i,j\right)<-2\cdot EL-1. \end{array}\right.$$

Note that in Equation (11), the difference values between −5 and 5, with a total of 11 bins, should be modified for data embedding. Another 11 empty bins should be prepared. The heights of bins with difference values between −5 and 5 result in the length of secret, S, at this stage.

In Figure 10b, with the embedding of the first stage, we have the image quality of 43.14 dB of PSNR, and 0.9813 of SSIM for marked image $X_{1}$. Embedding capacity reaches 181,188 bits for hiding into the original image at the size of 512×512. In order to make fair comparisons, we denote the capacity to be $\frac{181188}{512\times 512}=$0.6912 bit per pixel (bpp). For the embedding with Equation (11), a location map is not produced in Figure 10b, as we mentioned in Section 2.

By following in the same manner, in Figure 10c, we can rotate the marked image $X_{1}$ by 90°, train the weight factors of $\left(1,\;6,\;1,\;14\right)$, and perform data embedding to obtain $X_{2}$. The PSNR and SSIM correspond to the marked image quality of the marked image $X_{2}$, while the capacity denotes the accumulated capacity from the first two stages. We can successively rotate the marked image by 90°, train the weight factors, and perform data embedding. With this manner, results with the marked images $X_{3}$, $X_{4}$, and $X_{5}$ are presented in Figure 10d, Figure 10e, and Figure 10f, respectively. In each stage, no location map is produced.

**Figure 11:**
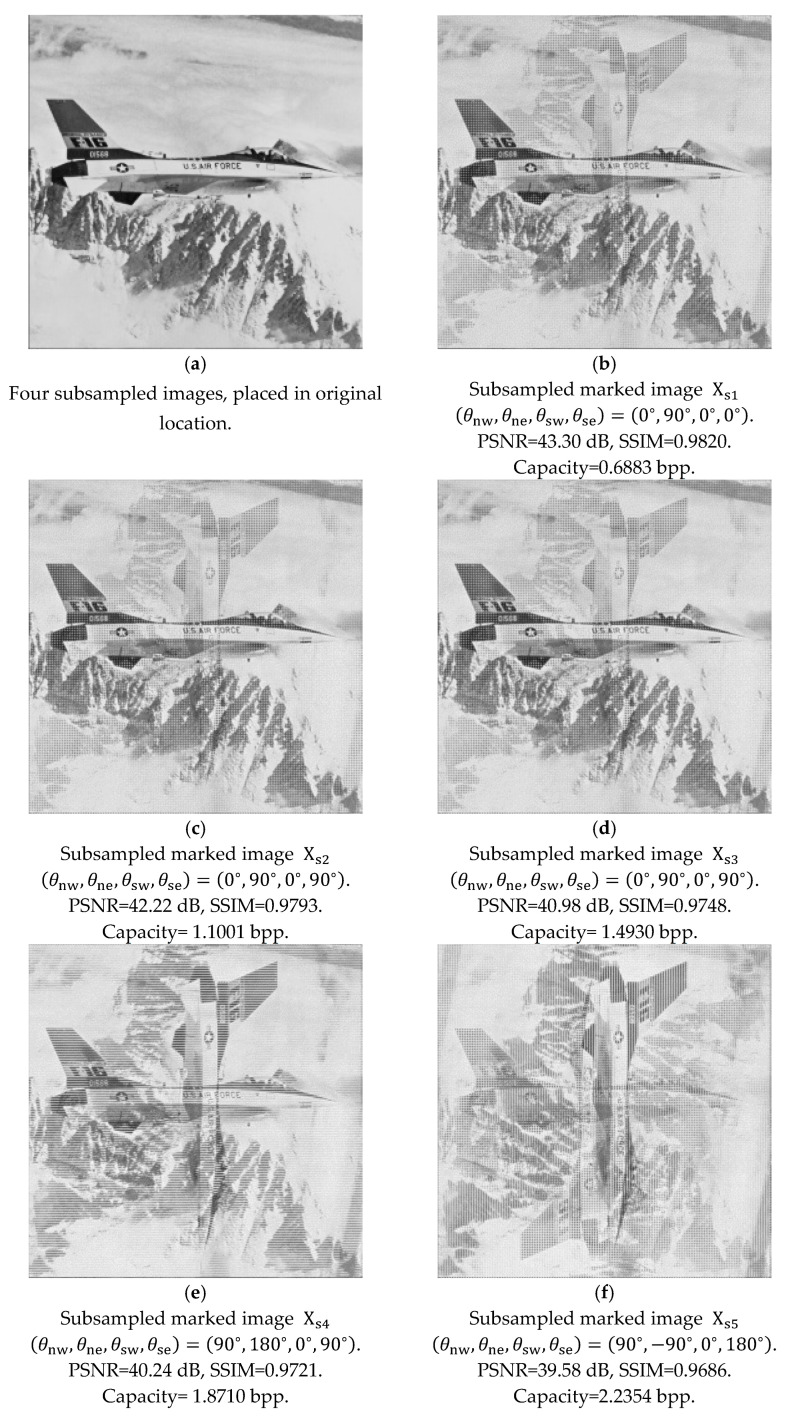
Illustration of data embedding with prediction-based scheme. (**a**) Original image X at size 512×512. (**b**–**f**) Intentional rotation of subsampled images for five stages.

Inspired by the approaches in Figure 10, we may apply a subsampling technique for reversible data hiding with the concept in Figure 6 and Figure 7. In Figure 11a, the original image F16 is sized at 512 × 512, and we intentionally place the pixels of four subsampled images in its original location, which is identical to Figure 10a. We can randomly rotate the four subsampled images, 256×256 each, by one of the four angles: namely, 0°, 90°, 180°, and −90°. The four independently rotated angles can be presented by $\left(\theta_{nw},\theta_{ne},\theta_{sw},\theta_{se}\right)$, corresponding to the four subsampled images, which may add more flexibility for reversible data hiding. By following the data-embedding procedures, in Figure 11b, four sets of weight factors, corresponding to four subsampled images, are chosen for the generation of predicted subsampled images. The subsampled image on the northeast (NE) side is rotated clockwise by 90°, represented by $\theta_{ne}=$ 90°. For the remaining three subsampled images on the northwest (NW), southwest (SW), and southeast (SE) sides, they are kept still; hence, $\theta_{nw}=\theta_{sw}=\theta_{se}=$ 0°. Secret information can be embedded accordingly, with the manipulation of four difference histograms corresponding to four subsampled images. The marked image $X_{s1}$ in Figure 11b, which looks blurred, is for demonstration purposes. Here, the subscript ‘s’ stands for subsampling. The PSNR and SSIM are calculated with the marked image by rotating the one on the northeast side back by 90°. This concludes the embedding of the first stage with subsampling and rotation. 

After completing the marked image of the first stage, we follow the same manner to obtain the four subsampled images in Figure 11b, and begin the procedures to acquire Figure 11c by rotating the subsampled images and embedding more secret information, with the condition that the number of stages can be determined by the user.

With our example, we choose to complete five stages. Again, Figure 11c is for demonstration purposes, and the PSNR and SSIM values are calculated with subsampled images rotated back to their predetermined locations. Figure 11d, Figure 11e, and Figure 11f also follow in this manner, with the provision of four sets of weight factors and four rotation angles of each stage, respectively. Finally, Figure 11f completes the five-stage data embedding.

Table 2 provides detailed statistics for embedding with five stages, corresponding to the marked images in Figure 11. In the second column in Table 2, ‘position’ denotes the position of the subsampled image. In the third column, ‘factors’ refer to weight factors $\left(w_{nw},\;w_{n},w_{ne},\;w_{w}\right)$ in Equation (3) which correspond to respective subsampled images at each stage. In the fourth column, ‘rotation’ denotes one of the four randomly designated angles corresponding to subsampled images. Predicted blocks can be generated after rotating back to their original positions after data embedding, and all the predicted blocks can be composed to become the predicted image. Then, embedding capacities are presented in the fifth column. The capacities of each stage denote the accumulated capacity. Next, the marked quality with PSNR and SSIM is shown in the sixth and seventh columns, and finally, correlation coefficients between the histogram of the original image and that of the marked one are displayed in the eighth column. 

In addition, when we check the increased capacity between two consecutive stages, we can find a reduction in the increased value of capacity as the embedding stages increase. In Figure 11 and Table 2, the increased capacities are lessened with the increase in embedding stages, while the errors induced after data embedding accumulate, leading to the reduction in PSNR values and the degradation of marked images.

We can compare results between the marked image quality and embedding capacity in Figure 10 and Figure 11. PSNR and capacity provide objective statistics, while SSIM denotes subjective measures. In Figure 11b,f, they have somewhat better PSNR values with a slightly inferior capacity measure. In Figure 11d,e, they have somewhat inferior PSNR values with a slightly better capacity measure. Figure 10c performs better than Figure 11c, with better PSNR and larger capacity. With a conventional prediction-based scheme, it produces the whole predicted image for data embedding, while with subsampling technique, four subsampled images perform embedding independently. Even when the marked pixels are placed back in their predetermined locations, subjective measures may become inferior; hence, the SSIM values imply this observation. The subsampling concept adds flexibility for the number of stages and rotation angles, which can be determined by users. To retain reversibility, these parameters play important roles for information security.

### 4.2. Example with Quadtree Decomposition

**Figure 12:**
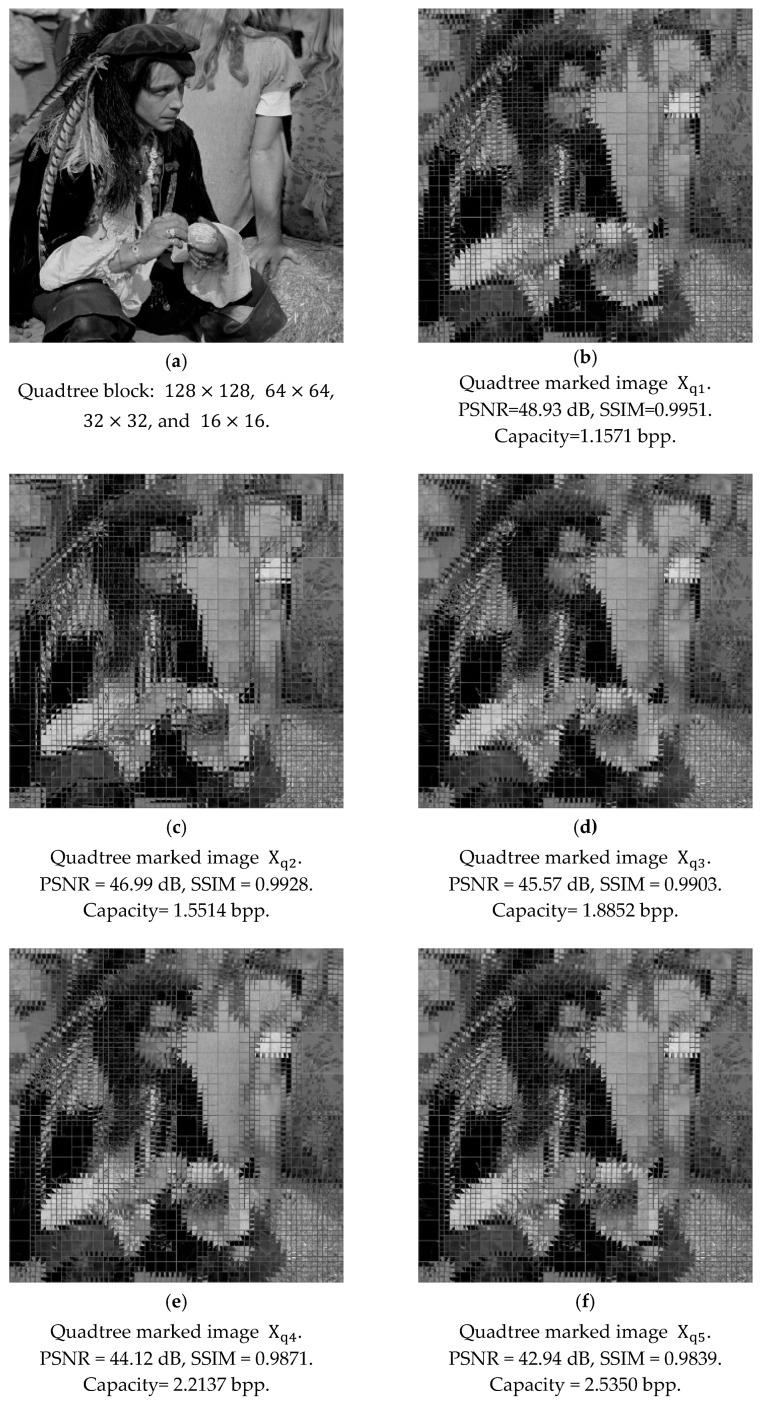
Illustration of data embedding with prediction-based scheme. (**a**) Original image X at size 1024×1024. (**b**–**f**) Intentional rotation of quadtree blocks for five stages.

In Figure 12, we present the example for reversible data hiding with quadtree decomposition with the block diagram in Figure 8. Figure 12a displays the original grayscale image male with a size of 1024×1024, and after quadtree decomposition, square blocks with sizes of 128×128, 64×64, 32×32, and 16 × 16 are formed, which follow the demonstration in Figure 8. For the first embedding stage, square blocks of the same size are intentionally rotated to one of the four designated angles, and the weight factors are trained for data embedding. After the prediction and embedding, blocks of the same size should be rotated back to their original place to obtain $X_{q1}$, where the subscript ‘q’ stands for quadtree decomposition. This concludes the embedding of the first stage with quadtree decomposition. Again, the marked image $X_{q1}$ in Figure 12b is for demonstration purposes, following its counterpart in Figure 11b. These data-embedding procedures follow those performed in Section 4.1. 

Next, we can follow the procedures above to complete the data embedding of the second stage. We train the weight factors and assign rotation angles, and subsequently embed data, following those in the first stage. Again, the marked image $X_{q2}$ in Figure 12c is for demonstration purposes. We observe the decrease in PSNR with the increase in capacity. In our simulations, we choose five stages for data embedding. The number of stages can be determined by users. By following in the same manner, we also carry out these procedures with the third, fourth, and fifth stages to obtain $X_{q3}$ in Figure 12d, $X_{q4}$ in Figure 12e, and $X_{q5}$ in Figure 12f, respectively. The capacities of each stage provided in Table 3 denote the accumulated capacity. After completing the embedding of the maximal capacity of each stage, corresponding PSNR values are calculated. The PSNR values imply the comparisons between the original image and its counterpart in the designated round, after data embedding. We present detailed statistics with the weight factors and rotation angles at all five stages in Table 3.

Additionally, when we check the increased capacity between two consecutive stages, we can find a reduction in the increased value of capacity as embedding rounds increase. In Figure 12 and Table 3, the increased capacities are lessened with the increase in embedding stages, while the errors induced after data embedding accumulate, leading to a reduction in PSNR values. With this observation, users may choose the appropriate number of embedding stages with the saturation of increased capacity. We also observe a similar phenomenon with the results in Section 4.1.

In addition to the marked quality and embedding capacity, weight factors and rotation angles with five rounds of embedding are displayed in Table 3. In the same stage, we can observe the variations in weight factors of different block sizes, due to inherent characteristics and rotation angles. In different stages, all the weight factors may behave differently due to data embedding and the assigned angles for rotation. Rotation angles are randomly appointed, in order to reach the flexibility for data embedding.

Finally, at the decoder, we need to look for the reversibility of the proposed algorithm, which implies the perfect separation of embedded data and the original image from the marked image. In order to examine the reversibility, we need to perform the above-mentioned procedures with a reverse manner for decoding. With the provision of weight factors and assigned rotation angles for decoding with $X_{q5}$, the embedded data at the fifth stage can be extracted, and $X_{q4}$ can be recovered. If we continue decoding in the same manner, the embedded data at the fourth stage can be extracted and $X_{q3}$ can be recovered. Continuing with this manner, we can successively gather the embedded data, and we can also obtain the original image X after decoding. After decoding one stage after another with the reverse approach, the extracted data and the original image can be recovered perfectly; hence, we can guarantee the reversibility of the proposed algorithm with quadtree decomposition. 

We may have a summary of the embedding capacity and the embedding stage. In Table 2 and Table 3, the multi-stage rotation method exhibits superior image quality preservation capabilities, achieving higher PSNR values than competing approaches at moderate embedding densities. This advantage stems from the progressive rotation mechanism that redistributes prediction errors across multiple stages, enabling the weighted average predictor to find optimal prediction parameters at each rotation angle. However, capacity accumulation follows a diminishing returns pattern, where each subsequent rotation stage contributes progressively less to embedding capacity, due to cumulative quality degradation. Users may choose a suitable number of stages for their own needs.

## 5. Simulation Results

In our simulations, we choose two test images, color image F16 at 512×512 and grayscale image male at 1024×1024, for performing reversible data hiding based on the demonstrations in Section 4. We employ data embedding with content characteristics using two aspects. First, we apply a prediction-based scheme to the original image, and then apply the subsampling method and bring about rotations for a few stages. Next, we employ the concept with quadtree decomposition for data hiding. We would like to look for performances between the embedding of different capacities and the marked image qualities. We also choose three other prediction techniques: namely, gradient adjacent prediction (GAP) [25], median edge detection (MED) [31], and rhombus prediction [32] for performing reversible data hiding. We compare the performances of all the techniques in this section.

### 5.1. Evaluations with Image Subsampling

By following the demonstrations in Section 4, we can perform data embedding with consecutive rotations of 90° of marked images, one stage after another. We can extend this concept with image subsampling. The original color image F16 is composed of three color planes: namely, red, green, and blue planes, as we mentioned in Equation (8).

**Figure 13:**
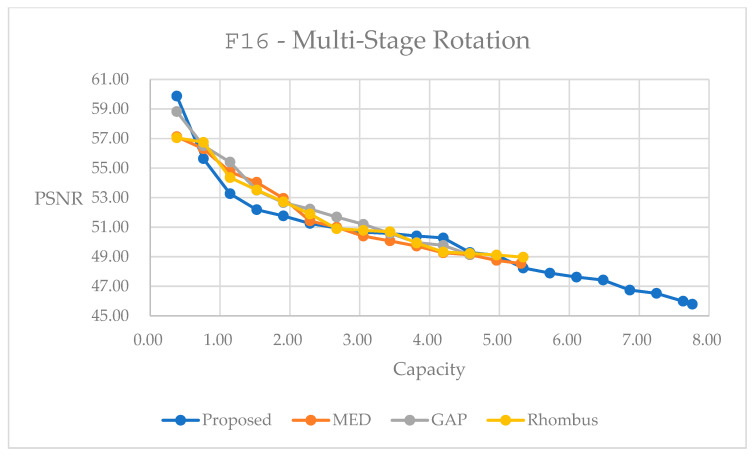
Performance comparisons for different predictors of color image F16, using multi-stage rotation method.

Figure 13 depicts the relationship between the marked quality in PSNR and the embedding capacity with the multi-stage rotation method, like the one presented in Figure 10. The weighted average prediction in Equation (3), and three other prediction methods for data embedding into color image F16 are compared. There is a trend that embedding more capacity leads to more errors induced, implying the quality degradation of marked image. With the multi-stage rotation method, we observe comparative or slightly degraded quality between the embedding range of 0.76 to 2.29 bpp to compare to other methods. More importantly, with the proposed method, we can embed a higher capacity. Data embedding is performed on the red, green, and blue planes successively, as depicted in the curves in Figure 13. In Table 4, we provide statistics with the largest embedding capacity for all methods. The multi-stage rotation method achieves a total embedding capacity of 7.7618 bpp while maintaining a PSNR of 45.79 dB, representing capacity improvements of 69.74% over GAP, 46.20% over MED, and 22.63% over Rhombus predictors. The subjective quality measure of SSIM, and the correlation coefficient between the original and marked histograms are also provided in Table 4.

**Figure 14:**
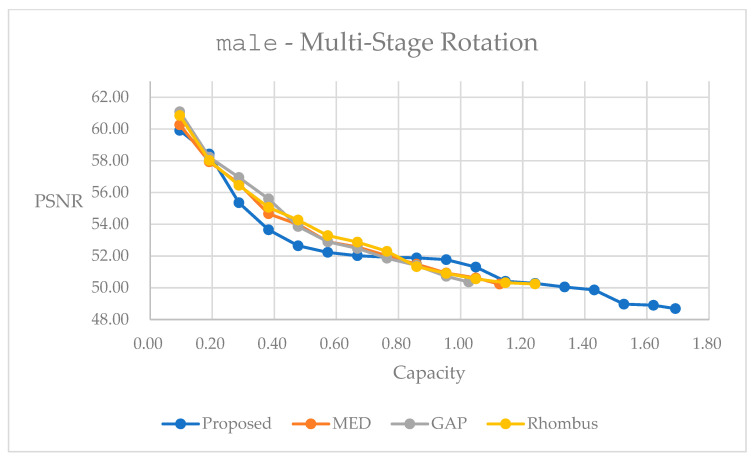
Performance comparisons for different predictors on grayscale image male, using multi-stage rotation method.

Figure 14 presents the relationship between the marked quality and embedding capacity for the grayscale image male by using the multi-stage rotation method. We observe similar trends between Figure 13 and Figure 14. We observe a somewhat inferior performance in quality within the embedding range of 0.2 to 0.8 bpp. Note that because we take the color image in Figure 13, we obtain a much higher capacity than its counterpart in Figure 14. In Table 5, we provide statistics with the largest embedding capacity for all of the methods. The multi-stage rotation method achieves a total embedding capacity of 1.6919 bpp while maintaining a PSNR of 48.69 dB, representing capacity improvements of 64.89% over GAP, 50.32% over MED, and 23.82% over Rhombus predictors.

The performance curves shown in Figure 13 and Figure 14 illustrate the superior embedding efficiency of the multi-stage rotation method across various embedding capacities. It consistently achieves higher PSNR values at low- and high-capacity levels, demonstrating better quality preservation during the embedding process. Notably, color images exhibit significantly higher embedding capacities due to data embedding into three color planes.

**Figure 15:**
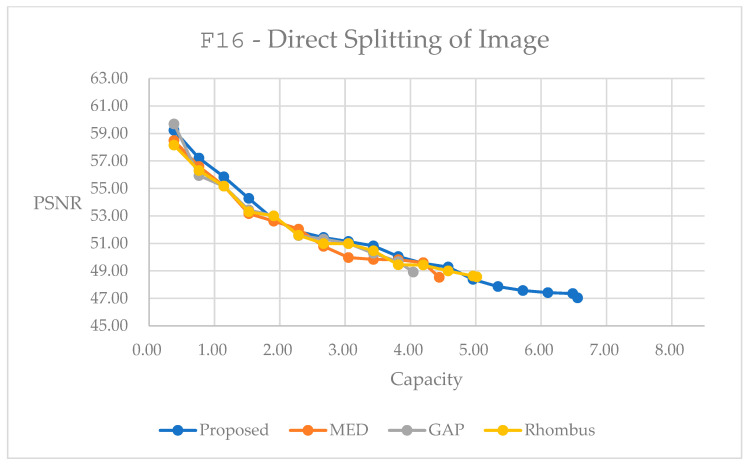
Performance comparisons for different predictors on color image F16 using direct splitting subsampling method.

**Figure 16:**
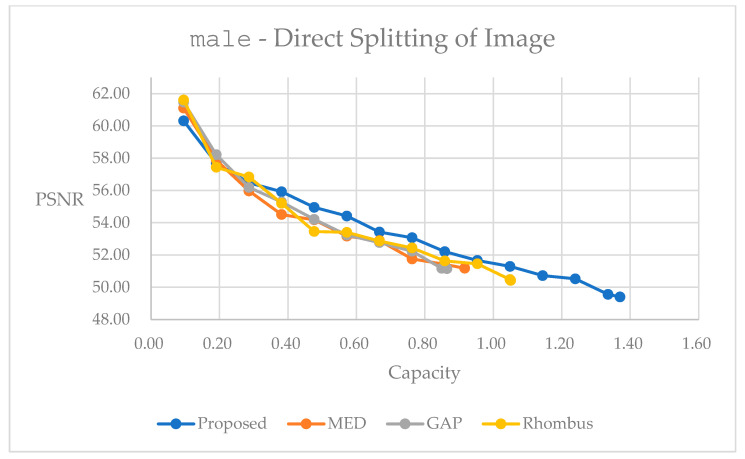
Performance comparisons for different predictors on grayscale image male using direct splitting subsampling method.

Then, we present simulations of the direct splitting method, which follows the concept in Figure 6. It partitions the original image into four quarter-sized blocks and performs a rotation with a designated angle to provide flexibility, and we can perform data embedding independently. In Figure 15, the proposed method performs generally better than the other methods. In Figure 16, it performs worse at the embedding range below 0.2 bpp. For all the other embedding ranges, the proposed method has its advantages in reversible data hiding. Table 6 and Table 7 present detailed statistics with the largest possible capacity for reversible data hiding. Hence, in Table 6, the subsampling and rotation method achieves a total embedding capacity of 6.5591 bpp while maintaining a PSNR of 47.03 dB, representing capacity improvements of 62.20% over GAP, 47.67% over MED, and 30.75% over Rhombus predictors for color image F16. Moreover, in Table 7, the subsampling and rotation method achieves a total embedding capacity of 1.3704 bpp while maintaining a PSNR of 49.40 dB, representing capacity improvements of 58.50% over GAP, 49.53% over MED, and 30.40% over Rhombus predictors for color image F16.

We can also make comparisons between Figure 13 and Figure 15 for F16, and Figure 14 and Figure 16 for male. We can easily observe that with the image splitting method, the proposed method performs better than that with the direct rotation of the whole image for data embedding. This may imply that image splitting may keep local characteristics in certain parts of the whole image, and hence, may have a better result for the predicted image. This may help the data embedding to be performed subsequently.

**Figure 17:**
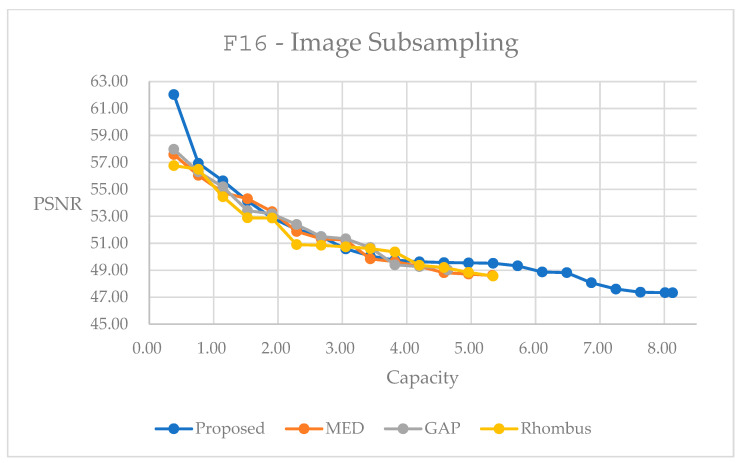
Performance comparisons for different predictors on color image F16, using block-based splitting subsampling method.

**Figure 18:**
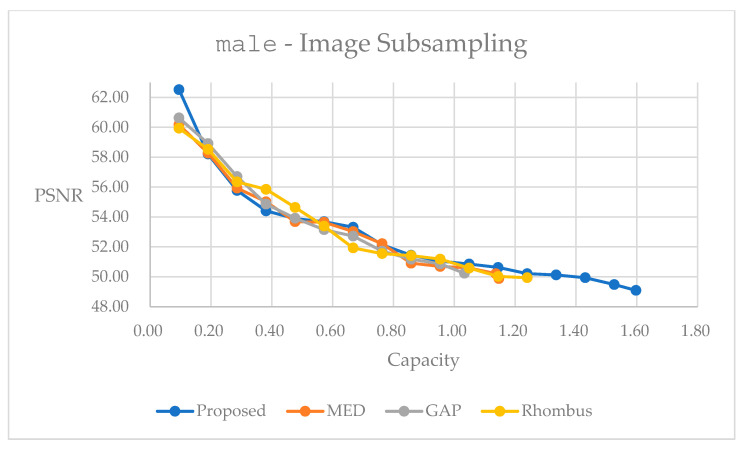
Performance comparisons for different predictors on grayscale image male, using block-based splitting subsampling method.

By following the concept in Figure 6 and Figure 7, we can apply subsampling for reversible data hiding. Figure 17 and Figure 18 present performance curves for the color image F16 and the grayscale image male, respectively. With our method, it performs generally better than others in Figure 17. In Figure 18, it performs worse in the embedding range between 0.2 and 0.7 bpp. With the proposed method, we can explore a much larger embedding capacity over those with the other methods. Users may look for the trade-off between embedding capacity and marked image quality. Table 8 and Table 9 present statistics with the largest possible capacity for reversible data hiding. Hence, in Table 8, the subsampling and rotation method achieves a total embedding capacity of 8.1287 bpp while maintaining a PSNR of 47.33 dB, representing capacity improvements of 75.30% over GAP, 52.51% over MED, and 30.05% over Rhombus predictors for color image F16. Comparatively, in Table 9, the subsampling and rotation method achieves a total embedding capacity of 1.5974 bpp while maintaining a PSNR of 49.09 dB, representing capacity improvements of 54.53% over GAP, 39.30% over MED, and 17.27% over Rhombus predictors for the color image F16.

The subsampling method provides the mechanism for ease in implementation. Its stable and predictable behavior across various image types makes it particularly suitable for scenarios requiring consistent performance. The splitting strategies reveal interesting trade-offs: block-based splitting achieves a superior capacity performance, particularly for color images with up to 24% improvement, but at the cost of slightly reduced quality preservation compared to direct splitting. Its primary limitation lies in its fixed partitioning strategy, which cannot adapt to local image content variations.

The performance curves shown in this section demonstrate the superiority of the weighted average prediction method along with block rotation over other methods. It denotes the advantage of maximum embedding capacity for both color and grayscale images. The inherent characteristics of the original image can be explored to help enhance the performances.

### 5.2. Evaluations with Quadtree Decomposition

The quadtree decomposition method implements content-adaptive block splitting based on local variance analysis, enabling optimized processing according to image complexity characteristics. It exploits image content heterogeneity, utilizing larger blocks in smooth regions for efficiency and smaller blocks in complex areas for quality preservation. Square blocks of different sizes can be produced with quadtree decomposition, and we may follow data-embedding techniques by serving each square block as a small image. This approach demonstrates the highest embedding capacity among the three proposed methods.

**Figure 19:**
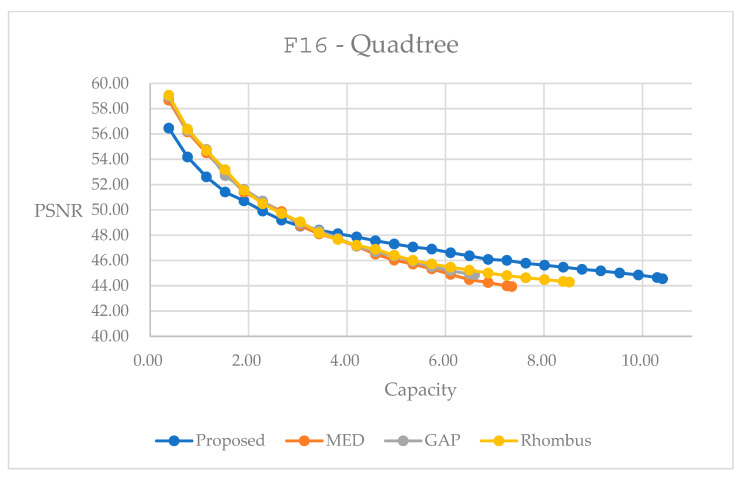
Performance comparisons for different predictors on color image F16 using quadtree decomposition method.

**Figure 20:**
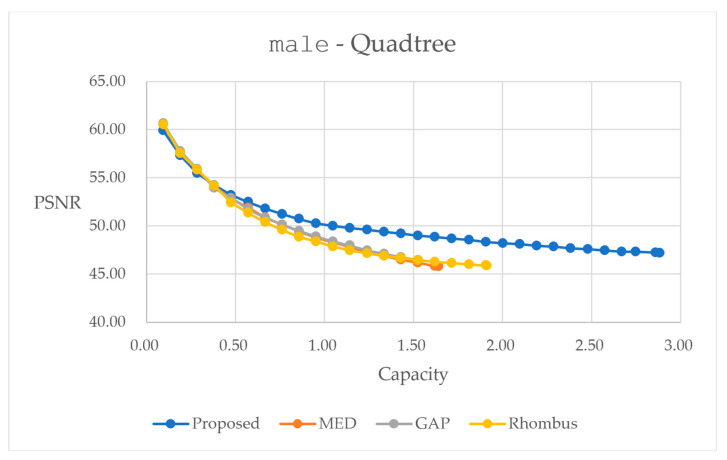
Performance comparisons for different predictors on grayscale image male using quadtree decomposition method.

Figure 19 and Figure 20 provide performance curves with quadtree decomposition. In Figure 19, for color image F16, the proposed method leads to better performances in the embedding range larger than 3.0 bpp. This might come from the fact that we apply quadtree decomposition to the red plane, and apply the composition of blocks to the green and blue planes with the consideration of easing implementation. Weight factors corresponding to different block sizes are trained with the red plane, and these weight factors directly apply to data embedding for all three color planes. Hence, there might be slight mismatches for the embedding into the green and blue planes. In contrast, when we assess the performance curves in Figure 20, we see that our method performs the best among all the prediction methods. This might be because we treat the grayscale image as the red plane in the color image. Weight factors are specifically trained for the grayscale image, and hence, we obtain enhanced results.

Table 10 and Table 11 present statistics with the largest possible capacity for reversible data hiding with quadtree decomposition. Hence, in Table 10, it achieves a total embedding capacity of 10.4090 bpp, while maintaining a PSNR of 44.55 dB, representing capacity improvements of 57.89% over GAP, 41.70% over MED, and 22.18% over Rhombus predictors for the color image F16. Moreover, in Table 11, it achieves a total embedding capacity of 2.8840 bpp while maintaining a PSNR of 47.20 dB, representing capacity improvements of 88.76% over GAP, 75.58% over MED, and 50.80% over Rhombus predictors for the color image F16.

The quadtree decomposition method achieves the highest embedding capacity among all tested approaches. The content-adaptive splitting strategy effectively exploits image heterogeneity, utilizing larger blocks in smooth regions while employing smaller blocks in complex areas. In addition, weight factors are trained with an exhaustive search to acquire an enhanced performance, based on inherent characteristics. However, this adaptability comes with increased complexity in parameter tuning in quadtree, which significantly affects the splitting behavior and subsequent embedding performance.

## 6. Conclusions

In this paper, we present a comprehensive reversible data-hiding framework that integrates the weighted average prediction with subsampling and quadtree decomposition by exploring the inherent characteristics of the original image. The proposed weighted average predictor utilizes the content adaptive properties for training suitable weight factors for reversible data hiding. We can divide the original image into blocks with subsampling and quadtree decomposition. Difference values between the original and predicted blocks are utilized for data embedding. To add the flexibility of reversible data hiding, rotations of blocks corresponding to different weight factors for prediction are employed. After prediction and data embedding, marked blocks are rotated back and combined to form the marked image with the utilization of inherent characteristics.

Simulation results validate the effectiveness of each embedding strategy for different application scenarios. With the proposed method, it can reach the largest embedding capacity to compare to other methods. Meanwhile, embedding a larger capacity leads to degraded quality in the marked image. Hence, users may look for the balance between embedding capacity and marked image quality from an objective viewpoint. When we examine the marked images subjectively, visual consistency with the presentation of structural similarity can be assessed. Most important of all, we can keep the reversibility of the proposed prediction method, along with three other prediction schemes.

### Limitations and Applicability

While the proposed method demonstrates a promising performance for natural images, it should be noted that its effectiveness relies on the presence of sufficient texture or intensity variation. For images that are nearly saturated, very dark, or dominated by large flat zones, the prediction-error histogram becomes highly concentrated. In such cases, the number of overflow locations is increased (thus, the location map is enlarged), and the visual impact of embedding tends to be more noticeable. Consequently, our approach may not be well suited to these extreme scenarios. Nevertheless, such conditions are rarely encountered in practical photographic acquisitions. Extending the scheme to handle these challenging cases, for example, by combining it with complementary RDH strategies, remains an interesting direction for future research.

In addition, the current design assumes that the cover image is square. The subsampling strategy requires equal-sized partitions, and the quadtree partitioning further requires the side length to be a power of two. For general rectangular images, the scheme can still be applied by selecting the largest inscribed square region and recording its top-left coordinate and side length as side information. This allows the decoder to locate the embedding region while maintaining the reversibility of the process. Extending the framework to fully support arbitrary image dimensions will be an interesting direction for future work.

## Figures and Tables

**Table 1:** Comparisons of different approaches in reversible data hiding.

Approach	Ref.	Advantage	Disadvantage
Histogram-based scheme	[2]	➢ Guaranteed quality, at least 48.13 dB	➢ Limited amount of capacity
Difference expansion scheme	[3]	➢ Enhanced capacity, at most 0.5 bpp	➢ Degraded quality ➢ Overflow problem
Conventional prediction and difference histogram scheme	[25,31,32]	➢ Combination of [2,3] ➢ Easy for generation of predicted image	➢ Little change for flexibility
Content-based prediction and difference histogram scheme	Proposed	➢ Combination of [2] and [3] ➢ Flexibility to meet different image characteristics	➢ Training for weights to produce prediction

**Table 2:** Prediction-based subsampling data hiding, with multi-stage rotation for grayscale image F16.

Stage	Position	Factors	Rotation	Capacity (bpp)	PSNR (dB)	SSIM	Hist. Corr.
1st	NW	(10,1,9,13)	0°	0.6883	43.30	0.9820	0.9480
	NE	(3,2,3,3)	90°				
	SW	(1,1,5,5)	0°				
	SE	(1,1,7,7)	0°				
2nd	NW	(4,1,3,9)	0°	1.1001	42.22	0.9793	0.9467
	NE	(6,9,8,6)	90°				
	SW	(6,3,7,7)	0°				
	SE	(1,1,3,6)	90°				
3rd	NW	(6,3,8,12)	0°	1.4930	40.98	0.9748	0.9393
	NE	(2,11,1,1)	90°				
	SW	(3,1,3,4)	0°				
	SE	(2,2,5,2)	90°				
4th	NW	(3,1,4,8)	90°	1.8710	40.24	0.9721	0.9180
	NE	(1,10,1,2)	180°				
	SW	(7,1,1,7)	0°				
	SE	(1,1,3,6)	90°				
5th	NW	(4,1,5,11)	90°	2.2354	39.58	0.9686	0.9022
	NE	(1,1,11,1)	−90°				
	SW	(5,7,6,7)	0°				
	SE	(1,1,4,2)	180°				

**Table 3:** Prediction-based quadtree data hiding with multi-stage rotation for grayscale image male.

Stage	Block Size	Factors	Rotation	Capacity (bpp)	PSNR (dB)	SSIM	Hist. Corr.
1st	128×128	(10,1,9,13)	90°	1.1571	48.93	0.9951	0.9663
	64×64	(3,2,3,3)	90°				
	32×32	(1,1,5,5)	−90°				
	16×16	(1,1,7,7)	90°				
2nd	128×128	(4,1,3,9)	180°	1.5514	46.99	0.9928	0.9530
	64×64	(6,9,8,6)	90°				
	32×32	(6,3,7,7)	−90°				
	16×16	(1,1,3,6)	180°				
3rd	128×128	(6,3,8,12)	0°	1.8852	45.57	0.9903	0.9294
	64×64	(2,11,1,1)	90°				
	32×32	(3,1,3,4)	−90°				
	16×16	(2,2,5,2)	90°				
4th	128×128	(3,1,4,8)	180°	2.2137	44.12	0.9871	0.9131
	64×64	(1,10,1,2)	90°				
	32×32	(7,1,1,7)	−90°				
	16×16	(1,1,3,6)	180°				
5th	128×128	(4,1,5,11)	180°	2.5350	42.94	0.9839	0.9009
	64×64	(1,1,11,1)	90°				
	32×32	(5,7,6,7)	180°				
	16×16	(1,1,4,2)	−90°				

**Table 4:** Multi-stage rotation with largest embedding capacity for color image F16.

Predictor	Capacity (bpp)	PSNR (dB)	SSIM	Hist Corr
Proposed	7.7618	45.79	0.9919	0.9954
GAP	4.5728	49.15	0.9938	0.9986
MED	5.3090	48.55	0.9936	0.9977
Rhombus	6.3292	47.72	0.9925	0.9972

**Table 5:** Multi-stage rotation with largest embedding capacity for grayscale image male.

Predictor	Capacity (bpp)	PSNR (dB)	SSIM	Hist Corr
Proposed	1.6919	48.69	0.9841	0.8917
GAP	1.0260	50.37	0.9937	0.9398
MED	1.1255	50.23	0.9947	0.9492
Rhombus	1.3664	48.76	0.9929	0.9698

**Table 6:** Direct splitting and rotation with largest embedding capacity for color image F16.

Predictor	Capacity (bpp)	PSNR (dB)	SSIM	Hist Corr
Proposed	6.5591	47.03	0.9795	0.9780
GAP	4.0438	48.91	0.9930	0.9985
MED	4.4417	48.53	0.9921	0.9984
Rhombus	5.0166	48.57	0.9917	0.9986

**Table 7:** Direct splitting and rotation with largest embedding capacity for grayscale image male.

Predictor	Capacity (bpp)	PSNR (dB)	SSIM	Hist Corr
Proposed	1.3704	49.40	0.9925	0.9303
GAP	0.8646	51.15	0.9951	0.9494
MED	0.9165	51.18	0.9958	0.9656
Rhombus	1.0509	50.42	0.9947	0.9846

**Table 8:** Subsampling and rotation with largest embedding capacity for color image F16.

Predictor	Capacity (bpp)	PSNR (dB)	SSIM	Hist Corr
Proposed	8.1287	47.33	0.9594	0.9633
GAP	4.6370	49.02	0.9938	0.9974
MED	5.3300	48.62	0.9936	0.9979
Rhombus	6.2505	48.90	0.9925	0.9968

**Table 9:** Subsampling and rotation with largest embedding capacity for grayscale image male.

Predictor	Capacity (bpp)	PSNR (dB)	SSIM	Hist Corr
Proposed	1.5974	49.09	0.9881	0.9063
GAP	1.0337	50.24	0.9932	0.9375
MED	1.1467	49.88	0.9933	0.9568
Rhombus	1.3622	49.29	0.9939	0.9715

**Table 10:** Quadtree decomposition and rotation with largest embedding capacity for color image F16.

Predictor	Capacity (bpp)	PSNR (dB)	SSIM	Hist Corr
Proposed	10.4090	44.55	0.9870	0.9934
GAP	6.5926	44.88	0.9887	0.9923
MED	7.3459	43.94	0.9883	0.9907
Rhombus	8.5193	44.28	0.9865	0.9942

**Table 11:** Quadtree decomposition and rotation with largest embedding capacity for grayscale image male.

Predictor	Capacity (bpp)	PSNR (dB)	SSIM	Hist Corr
Proposed	2.8840	47.20	0.9881	0.9450
GAP	1.5279	46.43	0.9869	0.9499
MED	1.6426	45.78	0.9865	0.9517
Rhombus	1.9125	45.88	0.9887	0.9884

## Data Availability

The original contributions presented in this study are included in the article. Further inquiries can be directed to the corresponding author.

## References

[1] Lombardi M., Pascale F., Santaniello D. (2021). Internet of Things: A General Overview between Architectures, Protocols and Applications. Information.

[2] Ni Z., Shi Y.-Q., Ansari N., Su W. (2006). Reversible data hiding. IEEE Trans. Circuits Syst. Video Technol..

[3] Tian J. (2003). Reversible data embedding using a difference expansion. IEEE Trans. Circuits Syst. Video Technol..

[4] Alattar A. (2004). Reversible Watermark Using the Difference Expansion of a Generalized Integer Transform. IEEE Trans. Image Process..

[5] Kim C., Yang C.-N., Leng L. (2025). Enhanced Dual Reversible Data Hiding Using Combined Approaches. Appl. Sci..

[6] Huang C.-T., Weng C.-Y., Shongwe N.S. (2023). Capacity-Raising Reversible Data Hiding Using Empirical Plus–Minus One in Dual Images. Mathematics.

[7] Kim C., Quero L.C., Jung K.-H., Leng L. (2024). Advanced Dual Reversible Data Hiding: A Focus on Modification Direction and Enhanced Least Significant Bit (LSB) Approaches. Appl. Sci..

[8] Khudhair S.K., Sahu M., K. R. R., Sahu A.K. (2023). Secure Reversible Data Hiding Using Block-Wise Histogram Shifting. Electronics.

[9] Xiong L., Han X., Yang C.-N., Shi Y.-Q. (2024). Reversible Data Hiding in Shared Images With Separate Cover Image Reconstruction and Secret Extraction. IEEE Trans. Cloud Comput..

[10] Yu C., Cheng S., Zhang X., Zhang X., Tang Z. (2024). Reversible Data Hiding in Shared JPEG Images. ACM Trans. Multimedia Comput. Commun. Appl..

[11] Zhang K., Yao H., Yang X., Qin C. (2025). Histogram Matching-Based Reversible Data Hiding for Intelligent Transportation Applications. IEEE Internet Things J..

[12] Wang X., Li X., Yang B., Guo Z. (2010). Efficient Generalized Integer Transform for Reversible Watermarking. IEEE Signal Process. Lett..

[13] Wang J., Chen X., Ni J., Mao N., Shi Y. (2019). Multiple Histograms-Based Reversible Data Hiding: Framework and Realization. IEEE Trans. Circuits Syst. Video Technol..

[14] Thodi D.M., Rodriguez J.J. (2007). Expansion Embedding Techniques for Reversible Watermarking. IEEE Trans. Image Process..

[15] Wu H.-T., Cao X., Jia R., Cheung Y.-M. (2022). Reversible Data Hiding With Brightness Preserving Contrast Enhancement by Two-Dimensional Histogram Modification. IEEE Trans. Circuits Syst. Video Technol..

[16] Zhou L., Han H., Wu H. (2021). Generalized Reversible Data Hiding with Content-Adaptive Operation and Fast Histogram Shifting Optimization. Entropy.

[17] Hou J., Ou B., Tian H., Qin Z. (2021). Reversible data hiding based on multiple histograms modification and deep neural networks. Signal Process. Image Commun..

[18] Arham A., Nugroho H.A. (2024). Enhanced reversible data hiding using difference expansion and modulus function with selective bit blocks in images. Cybersecurity.

[19] Wu F., Sun J., Zhang S., Xiong N., Zhong H. (2023). Efficient reversible data hiding via two layers of double-peak embedding. Inf. Sci..

[20] He W., Cai Z. (2021). Reversible Data Hiding Based on Dual Pairwise Prediction-Error Expansion. IEEE Trans. Image Process..

[21] Chang Q., Li X., Zhao Y., Ni R. (2021). Adaptive Pairwise Prediction-Error Expansion and Multiple Histograms Modification for Reversible Data Hiding. IEEE Trans. Circuits Syst. Video Technol..

[22] Wahed A., Nyeem H. (2022). Reversible data hiding with dual pixel-value-ordering and minimum prediction error expansion. PLoS ONE.

[23] Zhang T., Li X., Qi W., Guo Z. (2020). Location-Based PVO and Adaptive Pairwise Modification for Efficient Reversible Data Hiding. IEEE Trans. Inf. Forensics Secur..

[24] Kong X., He W., Cai Z. (2025). A Novel High-Fidelity Reversible Data Hiding Method Based on Adaptive Multi-pass Embedding. Mathematics.

[25] Chen Y.-H., Huang H.-C., Lin C.-C. (2015). Block-based reversible data hiding with multi-round estimation and difference alteration. Multimedia Tools Appl..

[26] Pan I.-H., Huang P.-S., Chang T.-J., Chen H.-H. (2022). Multilayer Reversible Information Hiding with Prediction-Error Expansion and Dynamic Threshold Analysis. Sensors.

[27] Huang H.-C., Fang W.-C. (2011). Authenticity Preservation with Histogram-Based Reversible Data Hiding and Quadtree Concepts. Sensors.

[28] Chang Q., Li X., Zhao Y. (2022). Reversible Data Hiding for Color Images Based on Adaptive Three-Dimensional Histogram Modification. IEEE Trans. Circuits Syst. Video Technol..

[29] Yang Y., Zou T., Huang G., Zhang W. (2021). A High Visual Quality Color Image Reversible Data Hiding Scheme Based on B-R-G Embedding Principle and CIEDE2000 Assessment Metric. IEEE Trans. Circuits Syst. Video Technol..

[30] Mao N., He H., Chen F., Zhu K. (2023). Reversible data hiding of color image based on channel unity embedding. Appl. Intell..

[31] Fan G., Pan Z., Gao E., Gao X., Zhang X. (2021). Reversible data hiding method based on combining IPVO with bias-added Rhombus predictor by multi-predictor mechanism. Signal Process..

[32] Lu S., Liao X., Mu N., Wu J., Le J. Reversible Data Hiding Based on Improved Rhombus Prediction Method. Proceedings of the 2019 Tenth International Conference on Intelligent Control and Information Processing (ICICIP).

